# The Flavor Chemistry of Fortified Wines—A Comprehensive Approach

**DOI:** 10.3390/foods10061239

**Published:** 2021-05-29

**Authors:** Teresa Abreu, Rosa Perestrelo, Matteo Bordiga, Monica Locatelli, Jean Daniel Coïsson, José S. Câmara

**Affiliations:** 1 CQM–Centro de Química da Madeira, Campus Universitário da Penteada, Universidade da Madeira, 9020-105 Funchal, Portugal; teresa.abreu@staff.uma.pt (T.A.); rmp@staff.uma.pt (R.P.); 2 Dipartimento di Scienze del Farmaco, Università degli Studi del Piemonte Orientale “A. Avogadro”, Largo Donegani 2, 28100 Novara, Italy; matteo.bordiga@uniupo.it (M.B.); monica.locatelli@uniupo.it (M.L.); jeandaniel.coisson@uniupo.it (J.D.C.); 3 Departamento de Química, Faculdade de Ciências Exatas e Engenharia, Campus da Penteada, Universidade da Madeira, 9020-105 Funchal, Portugal

**Keywords:** flavor, aroma origin, fortified wines, enology, vinification process, aging, odor descriptors, Madeira wines

## Abstract

For centuries, wine has had a fundamental role in the culture and habits of different civilizations. Amongst numerous wine types that involve specific winemaking processes, fortified wines possess an added value and are greatly honored worldwide. This review comprises the description of the most important characteristics of the main worldwide fortified wines—Madeira, Port, Sherry, Muscat, and Vermouth—structured in three parts. The first part briefly describes the chemistry of wine flavor, the origin of typical aroma (primary, secondary and tertiary), and the influencing parameters during the winemaking process. The second part describes some specificities of worldwide fortified wine, highlighting the volatile composition with particular emphasis on aroma compounds. The third part reports the volatile composition of the most important fortified wines, including the principal characteristics, vinification process, the evolution of volatile organic compounds (VOCs) during the aging processes, and the most important odor descriptors. Given the worldwide popularity and the economic relevance of fortified wines, much research should be done to better understand accurately the reactions and mechanisms that occur in different stages of winemaking, mainly during the oxidative and thermal aging.

## 1. A Short Introduction about Wine Flavor Chemistry

Chemically, wine is a fascinating and very complex matrix constituted by several hundreds of chemical compounds/groups—water, ethanol, glycerol, organic acids, carbohydrates, and, to a minor extent, terpenoids, pyrazines, higher alcohols, ethyl esters, fatty acids, nitrogenous compounds, sulphur compounds, furanic compounds, among others. These chemical groups were found in a broad range of concentrations (from a few mg/L to ng/L) and presenting different polarities and volatilities [1,2,3,4]. Table 1 shows the main volatile organic compounds (VOCs) found in wines. Some VOCs are responsible for the singular aromatic characteristics of some wine types. It should be mentioned that the most pleasant wines hardly have precise and simple to identify aromas; rather, they have complex aromatic aroma descriptors in which some fruit and floral perception is crucial, along with other spicy, woody, and/or toast notes. It should also be pointed out that the lack of aromatic faults or variations is also a constant and vital factor of quality [1,5].

The primary and secondary metabolites, which have been identified in grapes, musts, and wines, are synthesized throughout several pathways occurring from the vineyard to the consumer, including (i) biosynthesis in grapes; (ii) yeast metabolism during fermentative process; and (iii) several enzymatic and chemical reactions occurring during aging. Some of these metabolites actually contribute to the sensory perception of wine flavor and its specific organoleptic characteristics, being responsible for the wine aroma quality [1,2,3].

The final quality of wine depends on several factors and parameters, namely the grape varieties, geographical region, terroir and climatological conditions, the vinification process including fermentation conditions (must composition, dominant yeasts, pH, temperature), and aging. Terpenoids and their derivatives constitute significant markers of grape quality, contributing floral notes to the wine flavor and aroma when present in amounts higher than its odor threshold (OT) [12]. Nevertheless, several of the sensory properties that are commonly used to evaluate the quality of wine, including those that are deemed characteristic of the grape variety, are not commonly perceived in the grapes. They appear, mainly, through different chemical and biochemical pathways that occur during the winemaking process. The main key odorants and off-flavors, their impact on wine authenticity, and their evolution through aging, has been the focus of deep research and scientific interest. In addition, the interactions of odorants with other non-volatile wine compounds, and their effect on aroma quality, is also emerging as a potential field of curiosity in the scientific community. A deeper and comprehensive understanding of the biochemistry of grape-juice fermentation, and the chemistry of wine aging, is of utmost importance to help the wine industry by reinforcing the empirical knowledge of the traditional winemaker [13]. Different wine metabolomes can be used as useful tools in the research of key chemical components of a particular wine, allowing us to differentiate it from other wines [14,15,16,17]. In addition, they make it possible to connect chemical composition with the sensory properties, either by detecting influence VOCs or clarifying matrix effects.

The low concentration of key aroma compounds, such as esters, terpenoids, pyrazines, and thiols, in addition to the low odor threshold (OT) of many important contributors to the aroma, and the influence of alcohol matrix in the odor perception, make the accurate flavor definition of wine extremely challenging [5]. With the advance of analytical instrumentation, particularly the greater accessibility of gas chromatography-mass spectrometry (GC-MS), new insights about the flavor and its precursors in alcoholic beverages (e.g., wine) have been achieved [5,18,19,20].

To assess the impact of individual VOCs on the global flavor of wine, beverages, food, and other food-related samples, aroma extract dilution analysis (AEDA) [21,22,23] is one of the most appropriate and commonly-used procedures. However, the use of solvent-based extractions presents some drawbacks related to the loss of highly VOCs during the extraction and/or concentration of organic extract procedure, typically carried out under a nitrogen stream, in addition to the use of harmful organic solvents to both the operator and the environment. To overcome these shortcomings, emerging strategies of flavor dilution analysis, headspace solid-phase microextraction (HS-SPME) [1,24,25,26], and stir bar sorptive extraction (SBSE) [27,28,29] have been used with increasing frequency.

### The Wine Flavor: Origin and Influencing Parameters

**Figure 1:**
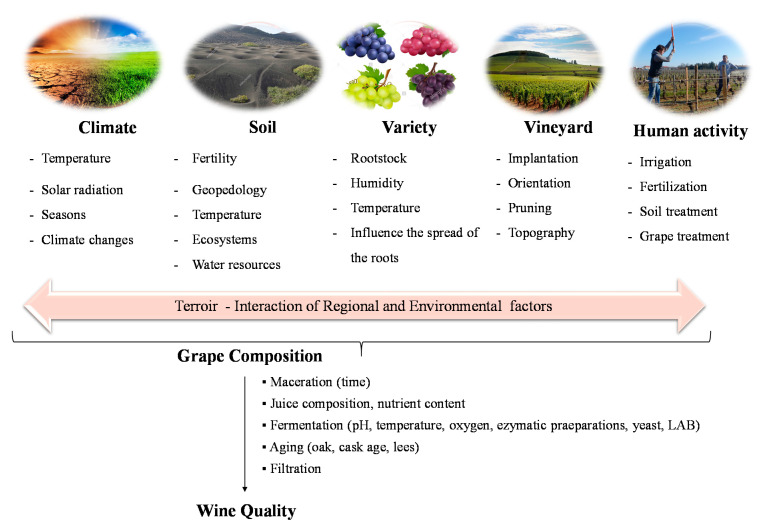
Most important influencing parameters on wine quality and aroma.

The flavor of wine is influenced by parameters including environmental factors (e.g., soil, climate), vineyard location, pre-fermentation biochemical phenomena (e.g., oxidations, hydrolysis), fermentation conditions (e.g., pH, temperature, microflora), vinification techniques, several post-fermentation processes (e.g., clarification, fining, filtration) to which the wine is submitted, and by the storage (humidity and temperature) and aging conditions (Figure 1) [17,18,19,20,21].

Based on their origin, the wine flavor can be categorized into (i) primary or varietal aroma, for compounds biosynthesized in grape; (ii) pre-fermentative aroma, formed during the processing of the grape harvest and subsequent operations; (iii) fermentative or secondary aroma synthesized during alcoholic fermentation by yeasts; and (iv) tertiary or aging aroma formed from enzymatic and physicochemical reactions such as oxidation and reduction, which occur during the conservation and wine aging [1,22].

The primary aroma is related to a complex set of compounds that can appear in free form, which contribute directly to odor (VOCs, odorants) and/or as odorless nonvolatile glycosides (glycosidically bound compounds) [1]. The acid and/or enzymatic hydrolysis of the glycosidically bound compounds may give raise to odorant aglycones during the winemaking process and aging, by the action of endogenous and/or exogenous glycosidase enzymes, or be caused by the moderate acidic conditions of grape juice and wine [23]. The primary aroma is biosynthesized during grape ripening. Their chemical nature and concentration depend on several parameters including soil, climate, phytotechnology and physiology of the vineyard, grape health status, and degree of ripeness of the grape. These types of aroma compounds contribute to the quality and aromatic typicity of young wines. Most of them are terpenoids (characteristic of Muscatel grapes and wines), C13-norisoprenoids (abundant in Chardonnay), and methoxypyrazines (characteristic of the Cabernet grapes and wines) [24,25]. In addition, some thiols have been identified as important contributors to the aroma of numerous grape varieties, namely Cabernet-Sauvignon, Sauvignon Blanc, and Merlot [26].

Pre-fermentative aroma, namely C6 aldehydes and C6 alcohols, are biosynthesized in the period between harvest and fermentation, due to the action of endogenous enzymes, as a result of the differentiated technological operations to which the grapes are submitted, namely harvesting, transport, crushing, and pressing. These operations allow the grapes enzymatic system interact with the membraneer lipids, releasing the polyunsaturated fatty acids—linoleic and linolenic acids [1,17,27], which, via oxidation, originate the C6 compounds. The concentration of C6 alcohols depends on grape variety, ripeness stage, treatment prior to fermentation, and temperature/duration of contact with skins [27]. The ratio (*E*)-3-hexenol/(*Z*)-3-hexenol may be useful for wine differentiation according to grape variety [17]. 

Fermentative aroma is produced by the action of yeast during alcoholic fermentation and by lactic bacteria if malolactic fermentation takes place. In the course of this process, the wines’ chemical and aromatic complexity increases significantly as a product of chemical transformations of the grape and must constituents, and as a result of the metabolism of the fermentative yeasts or bacteria (malolactic fermentation). Generally, the fermentation process is started and catalyzed by numerous indigenous yeast strains, namely *Saccharomyces*, *Kloeckera*, *Candida*, *Hansenula*, *Hanseniospora*, *Pichia*, and *Zigossacharimyce*. The yeast development during fermentation depends on must pH, fermentation temperature, nitrogen level, sulphur dioxide, and sugar content [19,28]. Besides ethanol and glycerol, several secondary metabolites are formed during the fermentation process that, while modest in quantity, can contribute significantly to the overall aroma of the wine. These include higher alcohols, fatty acids, and ethyl esters from volatile and fix acids, and, to a lesser extent, carbonyl compounds (e.g., aldehydes and ketones), lactones, and volatile phenols [1]. Malolactic fermentation is a secondary fermentation which takes place after alcoholic fermentation, and was generally performed in wines with enhanced acidity. The malolactic fermentation is conducted by malolactic bacteria (MLB), most often strains of *Oenococcus oeni*, and involves the decarboxylation of L-malic acid into L-lactic acid [29]. 

The compounds related to tertiary aroma arise during the wine maturation/conservation period. Throughout conservation, the volatile composition of the wine undergoes significant changes responsible for the evolution of the aroma, progressively losing the fruity and floral descriptors typical of young wines, and evolving to a more complex *bouquet* with caramel, dried fruit, spicy, toasty, and wood aroma notes. Some wines acquire their aromatic typicity after several years of aging, while others cannot stand long storage periods [30]. The excellence of the aging bouquet varies on wine origin (e.g., vineyard soil, microclimate), vintage, and diffusion of different compounds from oak to wine [21,31]. This diffusion process depends on the oak properties (e.g., geographic origin, oak species, seasoning of the staves, toasting, and cask age) [31,32]. The most common woods used during wine aging are obtained from different oak species such as *Quercus alba*, *Q. robur*, or *Q. petrea,* but also from other species known to contain high contents of ellagitannins, namely *Acacia*, *Castanea*, or *Prunus* [33]. The furanic compounds (e.g., 2-furfural, 5-hydroxymethylfurfural), lactones (e.g., sotolon, whiskylactone), and acetals (e.g., dioxane isomers, dioxolane isomers) are the chemical groups significantly associated to the evolution of wine ageing bouquet [20,34,35,36].

## 2. Fortified Wines

Fortified wines are characterized by a high alcohol content (between 15–22%, *v*/*v*), resulting from the addition of distilled spirits, usually a neutral grape spirit, and produced under oxidative conditions which determine the fortified wines’ typical flavor and aroma profile. This type of wine can be produced using both fermented (partially and/or totally) and unfermented grape must, according to EU regulation No. 479/2008. Following the intrinsic characteristic of the wines, they are usually classified as dessert, fortified, and generoso. Regarding the former, since these are generally consumed at the end of the meal, the definition of dessert wines reflects their widespread use. Regarding the second, due to their high alcohol content, these wines acquire the definition of fortified. Regarding the latter, the term generoso is relative to a significant alcohol and sugar content present in wines. Traditionally, fortified wines originate from Europe, however their production is spreading worldwide [37]. Although this spread is growing, some European countries remain the main producers [38]. Sherry, Port, Madeira, and Marsala are the most common types of fortified wines, whereas Vermouth (Italy), Moscatel de Setúbal (Portugal), and Commandaria (Cyprus), are produced in lower amounts.

### 2.1. Sherry Wine

**Figure 2:**
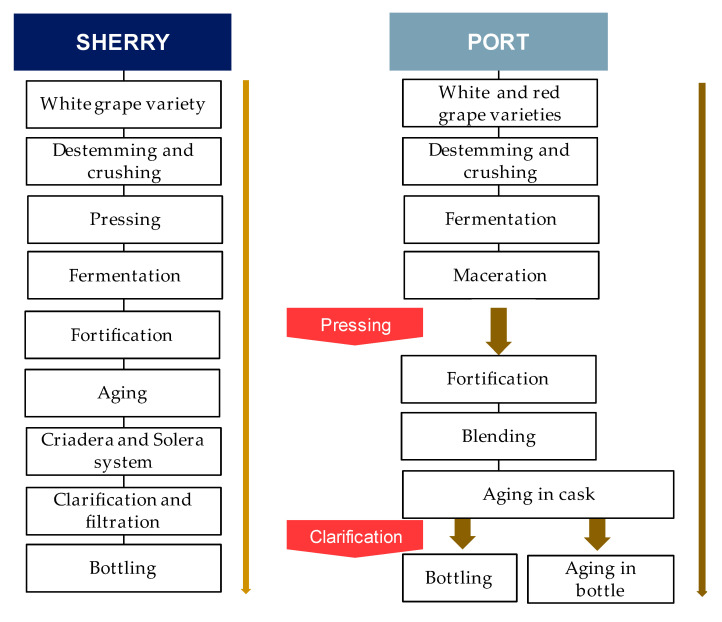
Sherry and Port’s winemaking processes.

Sherry (15–22% alcohol by volume) is produced in the province of Cádiz, Andalusia (south west Spain) by using white grapes cultivated in the Sherry triangle (Sanlúcar de Barrameda, Jerez de la Frontera and El Puerto de Santa Maria) [37,39,40]. The white grapes used are primarily from Palomino (both *Jerez* and *Fino*) however, Moscatel and Pedro Ximénez grapes are also utilized. Figure 2 reports the main winemaking steps for the production of Sherry wines. In the same way as those of still wines, the various winemaking steps follow this order: grape crushing, pressing, fermentation, racking, and the fortification step, followed by aging [40]. Once the base wine is ready, the wine is classified by professional tasters in order to define the most appropriate type of aging for each sample. Once classified, in order to prevent spoilage (e.g., acetic acid bacteria), the fortification step can begin. The addition of alcohol is defined according to the diverse types of aging the wines are going to receive. In detail, with an ethanol content set up to 15% (as in the case of *Fino* wine), the yeast cells are able to tolerate this percentage, thus maintaining the flor velum (biological aging). Conversely, if set to about 18% (as in the case of *Oloroso* wine), oxidative aging occurs, since the growth of flor yeasts is prevented [41].

**Figure 3:**
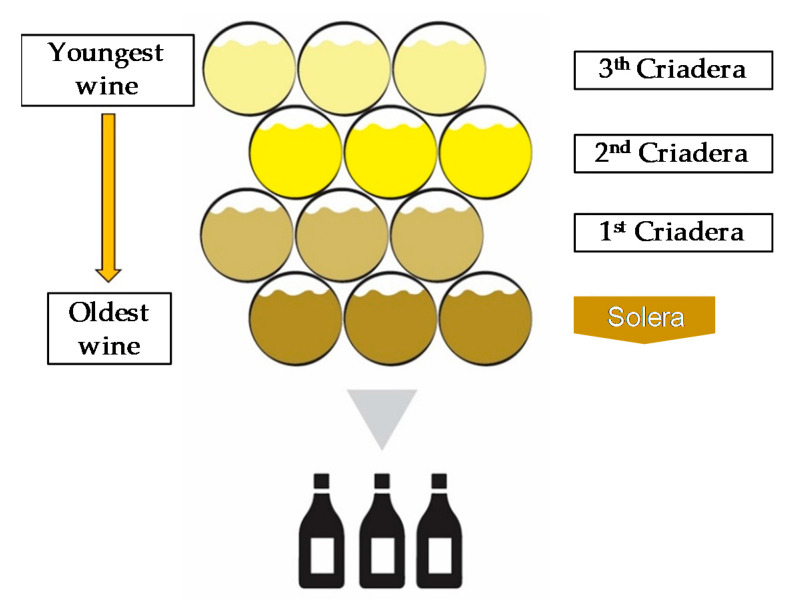
Sherry and Port’s winemaking processes.

Biological aging involves a static phase (“añadas”) during which the wine is kept in a butt for a variable number of years, followed by a dynamic one (“criaderas-solera”), consisting of a series of oak barrels in process of maturation. Nowadays, the solera system, established in the middle of the 19th century, is generally linked with modern Sherry production [42], however it is also utilized for other productions (e.g., Port, Madeira, brandy, and vinegar). In the initial stage, when the wine is young, about 15% (*v*/*v*) is defined as “sobretablas”. The middle stages are called “criaderas”, and the final one (also called “solera”) contains the oldest wine (Figure 3).

The *saca* process consists in removing a portion of wine from the solera by replacing it with an equal portion of younger wine from the first criadera. Generally, the volume of wine transferred ranges between 10–20% of the barrels capacity (about 500 L), even if, by law, up to 30% is allowed. The average wine age (from a minimum of 3 to 30 years) is determined by calculating the total system volume to annual volume removed ratio [41]. The solera system can be achieved through two different aging conditions: oxidative and biological [41]. Clarification represents the last phase before bottling. *Fino*, *Amontillado*, and *Oloroso* are the main styles of Sherry wines, characterized by certain peculiarities.

*Fino* is obtained by a gentle pressing of Palomino grapes; the wines appear fine and pale. The maturation period lasts between 3 and 8 years. Generally, the wines are left dry, characterized by a final alcoholic content ranging between 15.5–17% alcohol by volume [42,43]. *Fino* wines show a pale to light gold color, and they are characterized by a pungent apple flavor and peculiar notes such as fruity, floral, and cheesy [41,44]. 

Generally, *Oloroso*, produced using Palomino grapes obtained in hot climates, is darker, with a greater structure containing more phenols [42]. Among all Sherry styles, *Oloroso* is the most oxidized. The wines follow an oxidative aging process as their base wines are fortified up to 18% alcohol by volume. Its color varies from mahogany to amber, showing a complex bouquet characterized by distinct notes such as nutty, spicy, pungent smoke, tobacco, and leather [42,44]. Normally, the sweetest Sherries (about 20% alcohol by volume) are prepared starting from *Oloroso* wines by blending these with concentrated rectified must or naturally sweet wines [43].

*Amontillado* is characterized by a two-step process: (i) maturation under the flor velum (typical of *Finos*); (ii) oxidation period in the absence of flor [41]. From the organoleptic point of view, this wine is darker than *Fino* (ranging from amber to pale topaz). Thanks to the complexity of its bouquet, and due to the aging processes (*Fino* and *Oloroso*), *Amontillado* is the most valued Sherry style [45].

### 2.2. Port Wine

Port wine (15–22% alcohol by volume) is produced in the Douro Protected Designation of Origin (PDO) region (created and regulated in 1756). Lower Corgo, Upper Corgo, and Upper Douro represent the three distinct sub regions of the latter [46]. Apart from the Douro PDO region, other countries (e.g., Australia, South Africa, and the United States) produce Port-style wines by using techniques similar to those used in Portugal [47]. Portuguese varieties are commonly used in South Africa [46]. Among the many authorized grape cultivars, only six red ones are generally used to produce this kind of wine, namely Touriga Francesa, Tinto Cão, Touriga Nacional, Tinta Barocca, and Tinta Roriz [37,48]. Thanks to easy adaptation to the region’s soil characteristics, Touriga Franca represents half of the Douro PDO area. This variety shows a characteristic ruby color connected with complex notes such as cherry, raspberry, and blackberry [48]. Figure 2 schematized the main steps of Port winemaking. The fermentation temperatures of this type of wine range between 26–28 °C [38]. The singularity in Port production relies on the *aguardente* (wine spirit of 76–78% alcohol by volume; local definition relating to alcohol used in the fortification step). Once the amount of remaining sugars reaches the desired degree of sweetness (usually within 2–3 days), and having been evaluated by a panel for classification, the wines can begin the proper fortification phase [48]. According to the method of aging, wines can be assigned to two main categories: Ruby and Tawny style. The latter ages in smaller vessels (about 600 L; wooden) and the former in large ones (3–5 years followed by bottling). Tawny-style Ports show nutty, spicy, and woody notes, and specific brown and yellow hues. Ruby-style Ports show a red color, full-bodied structure, and fruity notes [38,49]. 

### 2.3. Madeira Wine

**Figure 4:**
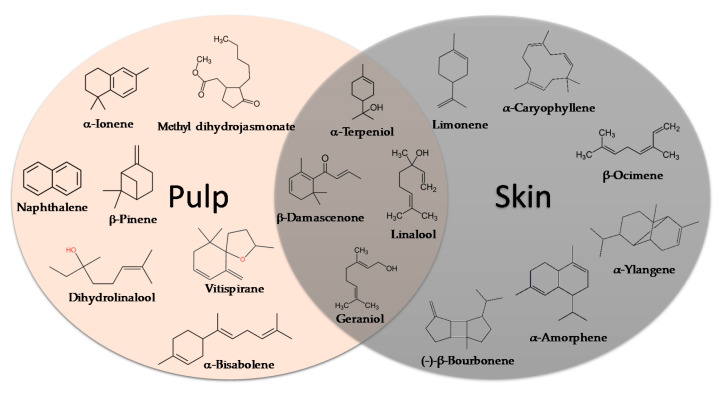
Some varietal compounds identified in pulp and skin of white *V. vinifera* L. grapes used in the production of Madeira wine [12,51].

Boal, Malvasia, Sercial, and Verdelho (white grape varieties), known as noble varieties, and Tinta Negra (TN, red grape variety) were the main *V. vinifera* L. grapes used in the production of Madeira wines. Some volatile compounds identified in the pulp and skin of these grape varieties are summarized in Figure 4. Tinta negra is the most predominant, constituting more than 80% of the vineyards, as it is very resistant to diseases (e.g., oidium, phylloxera) [50]. There are still other minor recommended (e.g., Bastardo, Verdelho Tinto) and authorized (e.g., Complexa, Deliciosa, Listrão) varieties to produce Madeira wine [50]. Terrantez and Bastardo are used rarely, even though they produce wines of distinctive quality. 

Grape volatile composition [51] is influenced by the presence of hundreds of VOCs involving different chemical groups, among which terpenoids and C13 norisoprenoids are the most representative for most of *V. vinifera* L. varieties [51]. These chemical groups were found to be dispersed between the pulp and skin of grapes, being in higher concentration in the skin than in the pulp [12]. Perestrelo et al. [12,51] recognized the varietal pattern of *V. vinifera* L. grape varieties from different geographical origins, and the results demonstrated that the amounts and number of VOCs in the skin are remarkably higher than those detected in the pulp. Moreover, different varietal volatile profiles were observed for the investigated grapes (Figure 4), independently of grape fraction. This difference could be explained by climatic factors (e.g., altitude, temperature, or humidity). Boal showed the highest levels of sesquiterpenic compounds, whereas Malvasia and Malvasia Cândida showed the highest level of monoterpenic compounds. 

According to Perestrelo et al. [52], the grape variety appears to have a remarkable influence in the sensorial characteristics of young Madeira wines, since unique aroma descriptors were linked to grape variety. Malvasia and Boal grapes are associated to almond and cocoa aroma descriptors, while Madeira wine obtained from Sercial and Verdelho grapes is associated to mushroom and honey descriptors [52]. 

**Figure 5:**
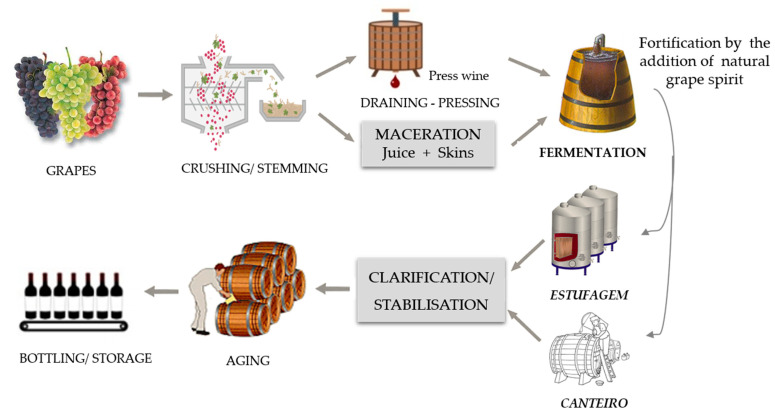
Schematic diagram of the winemaking process of Madeira wine.

The singular and unique characteristics of Madeira wines result from the particularities of the winemaking process (Figure 5). A natural grape spirit is added in order to stop the fermentation process and to obtain an ethanol content between 18 and 22% [1,35]. On the basis of the fermentation time, Madeira wine covers four wine types: dry (attained from Sercial, total sugars: 49.1 to 64.8 g/L), medium dry (Verdelho, 64.8 to 80.4 g/L), medium sweet (Boal, 80.4 to 96.1 g/L), and sweet (Malvasia, 96.1 to 150 g/L). Tinta Negra is used to produce all types of Madeira wines [1].

During the fermentation beyond ethanol, glycerol, and diols, several other fermentative-derived VOCs are produced by yeast metabolism, alcohols, esters, and acids being the most dominant. The predominant alcohols detected in Madeira wines are 3-Methylbutan-1-ol, 2-phenylethanol, 2-ethylhexan-1-ol, benzyl alcohol, and butan-2,3-diol [52,53,54]. Both 3-Methylbutan-1-ol and 2-phenylethanol have been reported to contribute positively to Madeira wine aroma with fruit, flower, and honey descriptors [4,52,54,55]. Furthermore, 2-ethylhexan-1-ol can explain the citrus descriptors of Madeira wines obtained for Malvasia, Sercial and Tina Negra grape varieties [52].

The ethyl esters of short- and medium-chain-length carboxylic acid (C_2_-C_10_) and acetates of short-chain-length alcohols (C_4_-C_6_) have been detected in Madeira wines at concentration higher than their OTs (few µg/L) [1]. These fermentative-derived VOCs contribute positively to Madeira wine aroma with fruity and/or floral aroma descriptors. Ethyl acetate, isoamyl acetate, ethyl hexanoate, and 2-phenyethyl acetate were the most significant esters for overall Madeira wine aroma. Additionally, the citrus descriptor characteristic of Malvasia, Sercial, and Tinta Negra young Madeira wines could be associated to the presence of hexyl acetate and ethyl 3-methylbutanoate [54,56].

Acetic acid is the predominant volatile acid in Madeira wines, indicating around 90% of total volatile acid fraction. When present at concentrations higher than 0.7 g/L, it contributes negatively with a vinegar-like descriptor to the wine aroma. Hexanoic, octanoic, and decanoic acids are the most predominant fatty acids found in Madeira wine [53,54]. The occurrence of these acids in wines at high concentrations is associated to negative aroma descriptors such as rancid, cheesy, and vinegar. In Madeira wines they normally occur at concentrations below their OTs [1].

### 2.4. Marsala Wine

This Italian wine is produced exclusively in the Marsala PDO region (the western part of Sicily Island). Marsala (15–20% alcohol by volume) is produced using Catarratto, Damaschino, and Grillo cultivars (white grapes), but red ones can be equally used to obtain ruby-colored wines (e.g., Pignatello, Perricone, or Calabrese) [37,57,58]. Although they are usually vinified as still wine, Marsalas are subject to significant pressing, thus obtaining higher amount of dry extracts (25–30 g/L) and oxidizable substances [58]. The fermentation is usually performed at a controlled temperature between 18–20 °C. According to the desired level of sweetness, the fortification step can be accomplished either during or after fermentation [57]. Fortification can be performed by using neutral grape spirit, brandy or mistelle (adding alcohol to unfermented grape juice or partially fermented wine). Marsala wines often maturate in wooden barrels in a similar way to the solera system. Marsalas are classified in dry, medium-dry, and sweet in relation to the degree of sweetness. 

### 2.5. Moscatel de Setúbal

This fortified wine, of about 17% alcohol by volume, is produced in the Setúbal PDO region, Portugal. The two traditional designations, “Moscatel Roxo” and “Moscatel de Setúbal”, are produced using at least 85% of the corresponding cultivars. In addition, there are other varieties which can be used for blending (e.g., Diagalves, Boais, or Arinto) [59]. A short fermentation period is characteristic of their vinification, which is subsequently interrupted (grape spirit fortification), followed by a period of maceration (prolonged contact between skins and fortified wine). According to the grapes used, Moscatel de Setúbal wines can be classified as white or red styles. Considering the aging time, wines are categorized as young or classic (up to 5 and more than 5 years, respectively).

### 2.6. Vermouth Wine

Vermouth is a particular type of fortified wine (15–21% alcohol by volume). Its main feature is the fact that this is also flavored by using spices, herbs, or their extract to a base wine [40,60]. Among the flavoring agents, wormwood, cloves, coriander, and chamomile are most commonly used. The fortification step begins after blending the base wine (preferentially neutral and produced from white grapes) with the extracts, and before aging. The period of maturation lasts an average of 4–5 years, although more aged Vermouth wines exist. Traditionally, Italian and French styles Vermouths are the most popular worldwide, however other types can also be found. The Italian Vermouths are sweet, showing an alcoholic content ranging between 15–17% alcohol by volume; conversely, the French ones are dry, reaching approximately 18% alcohol by volume. Generally, due to the contribution of the flavoring agents, Vermouths are characterized by a bitter aftertaste and a pleasant, intense flavor.

### 2.7. Commandaria Wine

This is the sweet wine par excellence of the island of Cyprus, produced in the Commandaria PDO region [61]. It is made by using Mavro and Négrette (sun-dried red) and Xynisteri (white) grape varieties. Due to the high levels of alcohol produced (approximately 15%), must fermentation is naturally ceased. Even if the fortification is not mandatory, the resulting wine can be fortified up to 20% alcohol by volume. Aging period is performed for at least 4 years in oak barrels following a solera-like system. These dark wines are characterized by honey and raisin notes.

## 3. Volatile Composition of Fortified Wines

### 3.1. Sherry and Port Wines

In a previous study, the key role of acetaldehyde in Sherry wines, proving to be a fundamental compound for their authenticity, has been reported [62]. Generally, acetaldehyde is abundant in fortified wines, and it is responsible for the pungent feature of *Finos* and for the ripe apple notes of Sherries. Acetaldehyde is also a precursor of other key odorants, such as 2,3-butanediol and acetoin (buttery notes), 1,1-diethoxyethane (green and fruity notes) and sotolon (nutty notes). In addition to these features, this compound is also responsible for the browning phenomenon that occurs in Sherry wines. Among the VOCs characteristic of *Fino* Sherry-type wines (biological aging) it is possible to find fruity notes due to ethyl octanoate (banana, pineapple), sotolon, acetaldehyde, 1,1-diethoxyethane, and ethyl acetate (pineapple). There are also spicy notes due to the compounds released by wood, (Z)-oak lactone (vanilla), eugenol and 4-ethylguaiacol (clove), and sotolon [63]. 

Other studies have highlighted the characteristic compounds of *Oloroso* wines (oxidative aging). Chemical, vegetable, fruity, balsamic, floral, and spicy tones represent the eight odorant series established for their description [64,65]. Considering *Oloroso* wines age between 0 and 14 years, ethyl butanoate (banana), sotolon, and ethyl octanoate were found to be the most active odorants. Acetaldehyde, 2,3-butanedione, and 1,1-diethoxyethane increased their impact during aging, rather than compounds from cask wood. If compared with *Fino*- and *Oloroso*-type wines, Amontillado showed that acetaldehyde, eugenol, and ethyl acetate were the compounds that most distinguish these three types of Sherry wines [66,67]. Significant changes in the aromatic profile of these wines were reported during the first years of the oxidative period. For Amontillado wines, it was concluded that the most representative odorant compounds are ethyl octanoate, ethyl butanoate, sotolon, eugenol, and ethyl isobutyrate. Another study, focused on the characterization of some hydroxy acids in different types of wines, including Sherries, showed that the latter had a concentration of these compounds between 2 and 40 times higher compared to all other samples [68]. The differences in concentration with respect to other types of wine are particularly significant in the case of 2-hydroxy-3-methylbutanoic acid and 2-hydroxy-2-methylbutanoic acid. This evidence might be related to the typical vinification and aging processes of Sherry wines. 

Considering Port wines, some studies focused on specific compounds formed during the Maillard reaction, particularly aldehydes and VOCs formed from carotenoid degradation. Another line of research has focused on the volatile compounds that form or vary during the aging process. Regarding the first group, for example, it has proven the strong link between β-carotene, lutein, and related compounds (β-damascenone, β-cyclocitral, and β-ionone), responsible for the sensorial impact of Port wines [69]. Interestingly, the branched aldehydes (dried fruit notes) and (*E*)-2-alkenals showed significant amounts in Ports (both Tawny and Ruby wines) [70]. In another study, the 3-deoxyosone content was evaluated during both the natural and forced aging samples. Interestingly, it emerged that this compound can be used as a wine aging marker due to the relationship between its yield and the reduction in sugars and amino acids in these wines during the Maillard reaction [71]. Due to the presence of high alcohol levels, Port wines showed high levels of VOCs extracted from wood (oak aging), in particular of oak lactone (β–methyl-γ-octalactone) [47]. It has been reported that one of the most significant aromatic compounds is sotolon. Both temperature and oxygen levels can influence the formation of sotolon (nutty and spicy notes) [72]. Older Ports showed less volatile sulphur compounds, such as 3-(methylthio)-1-propanol (cauliflower), 2-(methylthio)ethanol (French bean), and 3-(methylthio)-1-propionic acid (butter) when compared with young wines. Due to exposure to oxygen, aged Port wines show higher amounts of dimethyl sulphide, responsible for quince and truffle notes. In Tawnies, the aging process also influences the content of nor-isoprenoids, namely increasing vitispirane, 1,1,6-trimethyl-1,2-dihydronaphthalene (TDN) and 2,2,6-trimethylcyclohexanone (TCH). Similarly, the concentrations of β-ionone and β-damascenone are affected in vintage wines, and this behavior is related to oxygen exposure in bottle and cask aging [73].

### 3.2. Madeira Wine

**Figure 6:**
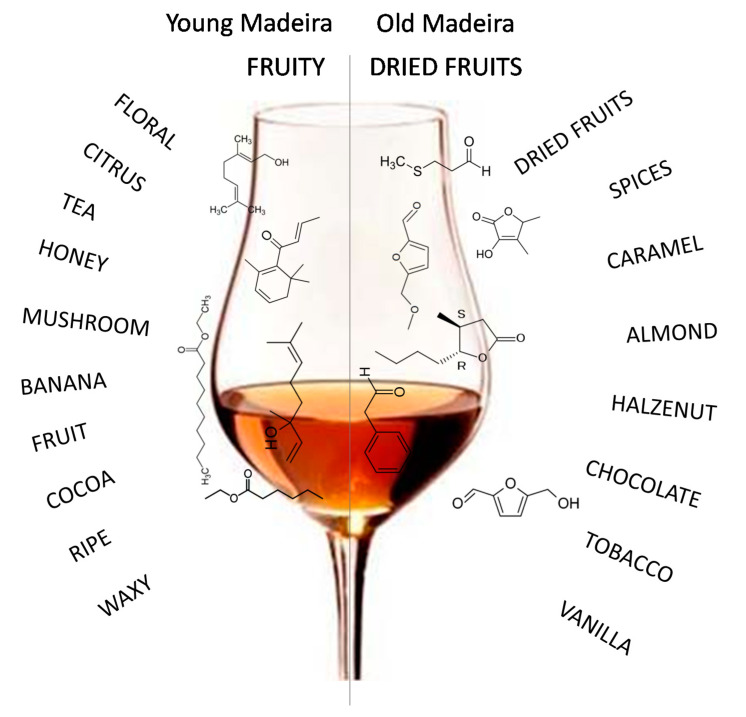
Characteristic aroma notes in young and old Madeira wines.

Highest quality Madeira wines are typically aged in *canteiro* (the wine barrels are placed under supports of wooden beams, called beds), which involve aging the wine in oak casks for least 3 years at temperatures ranging from 15 to 31 °C, in high humidity conditions (>70%), before being marketed. Nevertheless, a significant percentage of Madeira wines are submitted to the *estufagem* process, in which the wine is initially submitted to a thermal process for at least 3 months, during which the temperature gradually rises 5 °C/day and kept at 45–50 °C. After this period, the wines are submitted to a maturation step in oak casks for a least of 3 years before being marketed [74,75]. Madeira wine’s specific sensorial properties are strongly dependent on these production techniques, which are responsible for numerous reactions, mainly the Maillard reaction, Strecker degradation, and caramelization, influenced by a combination of numerous factors including pH, temperature, time, and dissolved oxygen and SO_2_, in addition to the diffusion of compounds (volatile substances and ellagitannins from the oak to the wine. The nature of the diffused compounds depends primarily on the oak properties (e.g., geographic origin, oak species, toasting degree, and oak age) [35]. Both enzymatic and non-enzymatic reactions occurring during winemaking are responsible for the main differences in the sensorial perception throughout the process. Pereira et al. [53] evaluated the behavior of the volatile pattern of Madeira wines submitted to the *estufagem* process (45 °C, 3 months) and to an overheating process (70 °C, 1 month). The obtained results indicated that accelerated aging favored the development of volatiles, such as phenylacetaldehyde, β-damascenone, and 5-(ethoxymethyl)-2-furfural. In addition, numerous varietal compounds (e.g., monoterpenic alcohols) responsible for the floral descriptors of some Madeira wines were not found after thermal treatment. Miranda et al. [75] evaluated the trend of acetic acid and ethyl acetate during the aging process of Madeira wine using both *estufagem* (forced aging associated with thermal treatment) and *canteiro* (aging in oak casks). The data indicated that these two VOCs showed a similar formation trend in both types of aging processes. More recently, Perestrelo et al. [54] evaluated the influence of sugar content, storage time (0, 1, and 4 months), and temperature (30, 45, and 55 °C) on volatile profile of Tinta Negra wines. A total of 65 VOCs were identified, from which 14 appear during storage. This result suggested that storage promotes the overall aroma of Tinta Negra wines. Additionally, during the aging process the wines acquired aroma descriptors (e.g., caramel, dried fruit, spice, toast, and wood) as a result of the Maillard reaction, Strecker degradation, caramelization, and microbial activity, whereas lost their fresh, floral, and fruity descriptors characteristic of younger wines (Figure 6). The results obtained in this research represent a useful tool to promote alterations in the *estufagem* process, as well as assess the effect of storage on volatile profile.

Wine volatile pattern represents a significant role in wine quality, as its volatiles endorse numerous sensations through wine drink, odors, and tastes that lead to consumer acceptance and/or rejection. Overall, an occurrence of a single molecule, at a concentration higher than its OT, is sufficient to deliver a distinctive aroma (key odorant). Nonetheless, the key odorant comprises of a mixture of hundreds of diverse odorants (VOCs). Numerous VOCs are present in only trace levels (frequently from μg/L or ng/L level), but, even at concentrations lower their OT, they may donate to the global aroma; due to their combinations with other VOCs, they can consequently have an influence on wine aroma perception [1,52,76]. Moreover, several investigations have centered on the identification of putative key odorants that can provide independently to the overall Madeira wine aroma [4,77]. Campo et al. [4] established the aroma pattern of four Madeira wines from the grape varieties Malvasia, Boal, Verdelho, and Sercial. The gas chromatography-olfactometry (GC–O) profile of Madeira wines lack the most significant varietal aromas, such as linalool, 3-mercaptohexyl acetate, and methoxypyrazines, whereas are rich in sotolon, phenylacetaldehyde, and wood extractable aromas. They also contain a great number of powerful odorants not recognized, which were not even found in the matching young wines. Moreover, furanic compounds, such as 2-furfural, 5-methyl-2-furfural, 5-hydroxymethyl-2-furfural, and 5-(ethoxymethyl)-2-furfural are quantitatively significant in Madeira wines analyzed, but they were not identified by GC–O, which indicate that furanic compounds probably do not contribute significantly to Madeira wine aroma due to their high OT. In addition, the highest scores detected were related to candy, toasty, woody, and dried fruit descriptors. Nevertheless, these depend on grape variety, winemaking, and the aging process. Silva et al. [77] evaluated the effect of forced-aging on Madeira wine aroma by GC-O. Several Maillard by-products, such as sotolon, 2-furfural, 5-methyl-2-furfural, 5-(ethoxymethyl)-2-furfural, methional, and phenylacetaldehyde were detected, which clarify the baked, brown sugar, and nutty aroma descriptors of Madeira wine. From these studies [4,77], sotolon, due to its low OT (10 μg/L), is reported as a key odorant on the conventional aroma of aged Madeira wines [36,78,79]. The formation pathways of sotolon are not well elucidated [79], although many precursors and pathways have been suggested for its formation in wines, namely oxidative degradation of ascorbic acid in the presence of ethanol, aldol condensation, peroxidation of acetaldehyde, and the Maillard reaction, among others [36,78]. Nevertheless, it is documented that the sugar content, wine oxidation, storage time, and temperature are strongly linked to sotolon formation [78,80]. According to Câmara et al. [36], Malvasia has a higher level of sotolon when compared with Sercial, and the study observed that sotolon amount is dependent of aging, sugar content, and furanic compounds. Some lactones derived from oak, such as γ-butyrolactone, pantolactone, *cis* and *trans*-whisky lactone, represent important odorants in Madeira wines aged in oak casks. During the aging process in oak casks, their concentration tends to increase notably [20].

## 4. Conclusions

The current review is focused on the most worldwide fortified wines—Madeira, Port, Sherry, Vermouth and Muscat—which are produced using traditional and particular vinification procedures. The distinctive odor descriptors of these fortified wines result from the integration of different factors such as grape varieties, geographical region, vinification, and aging processes. VOCs are formed in different ways and in the different stages that occur; from the formation of the grapes to when the final consumer–terpenoids, C13 norisoprenoids, and sesquiterpenoids are biosynthesized in the grapes, alcohols, esters, and acids are the result of the microorganisms actions, while lactones, furanic compounds acetals, and volatile phenols, are synthesized during aging. Nevertheless, the impact of these VOCs to the global aroma of fortified wines depends on their concentration and OTs. According to previous studies, reported in the current review, during aging the fortified wines lose their freshness and fruitiness odor descriptors (primary and secondary aromas) and other odor descriptors are formed, namely spicy, caramel, toast, wood, and dried fruits, as result of the Maillard reaction and diffusion from the oak to the wines. Sotolon, oak lactones, 2-furfural, 5-methyl-2-furfural, 5-(ethoxymethyl)-2-furfural, and 5-phenylacetaldehyde are some VOCs that could explain the odor descriptors related to wine aging.

## Figures and Tables

**Table 1:** Some important volatile organic compounds (VOCs) identified in fortified wines, chemical structure, aroma descriptors, and odor threshold (OT) [4,5,6,7,8,9,10,11].

Chemical Groups	VOCs	Chemical Structure	Aroma Descriptor	OT ^1^ (µg/L)
Terpenoids	α-Terpeniol	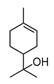	Floral, linden	250
	Linalool	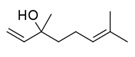	Rose	25
	Geraniol	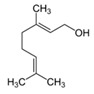	Citrus, floral	20
	Citronellol	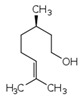	Citrus, floral, sweet	100
	Rotundone	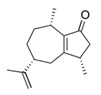	Pepper, spicy	0.016
C13 Norisoprenoids	β-Damascenone	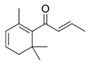	Boiled apple, sweet	0.05
	2,6,6-Trimethylcyclohex-2-ene-1,4-dione	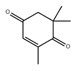	Musty, citrus, sweet honey	25
	TDN ^2^	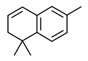	Floral, fruit	4
	Vitispirane	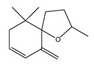	Floral, spice, wood	800
C6 compounds	1-Hexanol	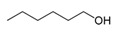	Herbaceous	8000
	(Z)-3-Hexen-1-ol	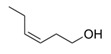	Green, bitter	400
	1-Hexanal	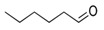	Green	97
Higher alcohols	3-Methylbutanol	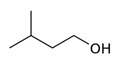	Fusel, sour	30,000
	2-Phenylethanol	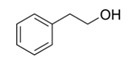	Rose, honey	140,000
	Benzyl alcohol	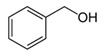	Blackberry	200,000
Ethyl esters	Ethyl acetate	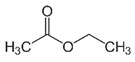	Solvent, nail polish, fruity	12,000
	Ethyl hexanoate	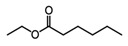	Green apple	14
	Ethyl octanoate	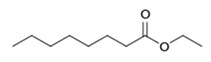	Sweet, flower	2
	Ethyl decanoate	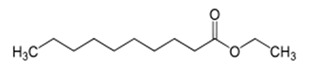	Brandy, grape	200
	Ethyl lactate	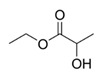	Butter	150,000
	Diethyl succinate	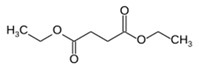	Melon	500,000
Acetates	Isoamyl acetate	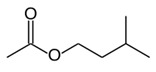	Banana, sweet	160
	2-Phenylethyl acetate	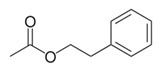	Rose, flower	1800
Acids	Acetic acid	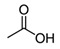	Vinegar, sour	200,000
	Hexanoic acid	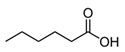	Cheese, fatty	3000
	Octanoic acid	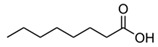	Fatty, rancid	10,000
	Decanoic acid	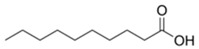	Fatty, rancid	15,000
Carbonyl compounds	2-Phenylacetaldehyde	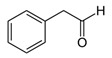	Floral	1
	Diacetyl	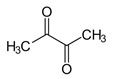	Buttery	100
	Benzaldehyde	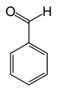	Almond	2000
Furanic compounds	2-Furfural	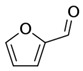	Wood, nut	14,100
	5-Methyl-2-furfural	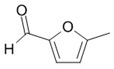	Caramel	20,000
	HMF ^3^	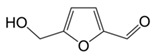	Almond, nut	10,000
Volatile phenols	4-Vinyl-guaiacol	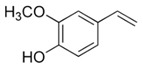	Smoke, phenolic	40
	Methyl vanillate	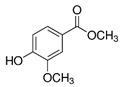	Vanillin	3000
	4-Vinyl-phenol	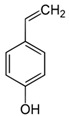	Spicy, pharmaceutical	180
Lactones	Sotolon	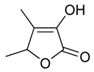	Wood, nut, toast	19
	γ-Decalactone	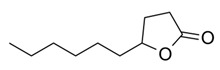	Peach	88
	Whisky lactone	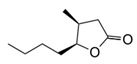	Caramel, nut, toast	67
Pyrazines	IBMP ^4^	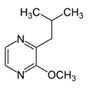	Leafy	0.016
	SBMP ^5^	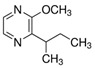	Green, pepper	0.002
	IPMP ^6^	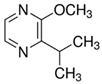	Leafy	0.002
Sulphur compounds	3-Mercaptohexyl acetate	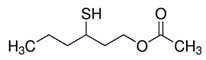	Passion fruit, box tree	0.0042
	4-(Methylthio)-4-methyl-2-pentanone	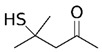	Box tree, tropical fruit	0.0008
	3-Mercaptohexanol	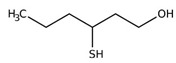	Passion fruit, grapefruit	0.06

^1^ OT—Odor threshold was determined using a synthetic wine model with an ethanol content ranging from 10 to 12% of ethanol; **^2^** TDN: 1,2-dihydro-1,1,6-trimethylnaphthalene; ^3^ HMF-5-hydroxymethyl-2-furfural; ^4^ IBMP: 3-isobutyl-2-methoxypyrazine; ^5^ SBMP: 3-sec-butyl-2-methoxypyrazine; and ^6^ IPMP: 3-isopropryl-2-methoxypyrazine.
